# A successful intraoperative diagnosis of coexisting lymphoma and endometrial cancer

**DOI:** 10.1186/s12957-019-1708-3

**Published:** 2019-10-07

**Authors:** Ai Yoshino, Eiji Kobayashi, Mayu Shiomi, Kazuaki Sato, Michiko Ichii, Yutaka Ueda, Tadashi Kimura

**Affiliations:** 1 Department of Obstetrics and Gynecology, Osaka University Graduate School of Medicine, 2-2, Yamadaoka, Suita, Osaka, 565-0871 Japan; 2 Department of Pathology, Osaka University Graduate School of Medicine, 2-2, Yamadaoka, Suita, Osaka, 565-0871 Japan; 3 Department of Hematology and Oncology, Osaka University Graduate School of Medicine, 2-2, Yamadaoka, Suita, Osaka, 565-0871 Japan

**Keywords:** Endometrial cancer, Lymphoma, Multiple malignancies, Synchronous carcinoma

## Abstract

**Background:**

The coexistence of hematological malignancy with endometrial cancer is a rare phenomenon. We report a case of coexistence of endometrial cancer with follicular lymphoma which we suspected preoperatively and diagnosed during surgery by a multidisciplinary intraoperative assessment.

**Case presentation:**

A 67-year-old woman was referred to our hospital due to a suspicion of an endometrial cancer. Endometrial biopsy revealed grade 1 endometrioid adenocarcinoma. MRI showed invasion of the tumor into the outer half of the myometrium, and abdominal CT showed para-aortic and atypical mesentery lymphadenopathy which was suspected to be metastasis of endometrial cancer or malignant lymphoma. Abdominal hysterectomy with bilateral salpingo-oophorectomy, pelvic and para-aortic lymphadenectomy, partial omentectomy, and mesentery lymph node biopsy for endometrial cancer were performed. The mesentery and para-aortic lymph nodes that were sent for frozen section analysis showed no metastasis of the endometrial cancer. We simultaneously conducted an unusual intraoperative emergent four-color flow cytometry and intraoperatively diagnosed a B cell lymphoma in the mesenteric lymph nodes. Because this multidisciplinary assessment, we were able to avoid an unnecessary intestinal resection. The final pathological diagnosis was an endometrioid carcinoma (G1, FIGO stage IA), with a synchronous follicular lymphoma.

**Conclusion:**

Although a rare event in endometrial cancer surgery, it is necessary to be alert to the possibility of a synchronous lymphoma in cases of unusual site adenopathy.

## Background

When multiple primary malignancies are present at the time of diagnosis, they are classified as either synchronous or metachronous primary tumors. Synchronous multiple primary malignancies are defined as two or more primary tumors that are each diagnosed at an interval of less than 6 months apart. In contrast, metachronous multiple primary malignancies are defined as two or more primary tumors that are diagnosed at an interval of more than 6 months apart [1]. The vast majority of patients have multiple metachronous malignancies, whereas synchronous primary tumors are rare, ranging from 1.2–5.1% of cases at autopsy and in clinical studies [2–4] .Furthermore, the coexistence of a hematological malignancy with a gynecologic neoplasm, especially an endometrial cancer, is an ultra-rare phenomenon.

There have only been a few reports of cases of synchronous hematological malignancies with endometrial cancer. In general, the presence of another malignancy is an unexpected finding during surgery for endometrial cancer, and its diagnosis has usually only been made incidentally after staging surgery [5–10]. We herein report a case of the coexistence of an endometrial cancer accompanying a follicular lymphoma, which we suspected preoperatively. This is the first report of a case of a synchronous endometrial cancer with a malignant lymphoma which was diagnosed during surgery by a multidisciplinary intraoperative assessment.

## Case presentation

A 67-year-old woman (gravida-3, para-2, menopause at age 50) was referred to our hospital due to a suspicion of an endometrial cancer detected at regular follow-up. She had neither typical complications nor a family history for the disease. She exhibited no indicative symptoms, including uterine bleeding, pelvic pain, night sweats, weight loss, or fever. She did have a medical history of a cervical spine injury 2 years prior.

**Fig. 1:**
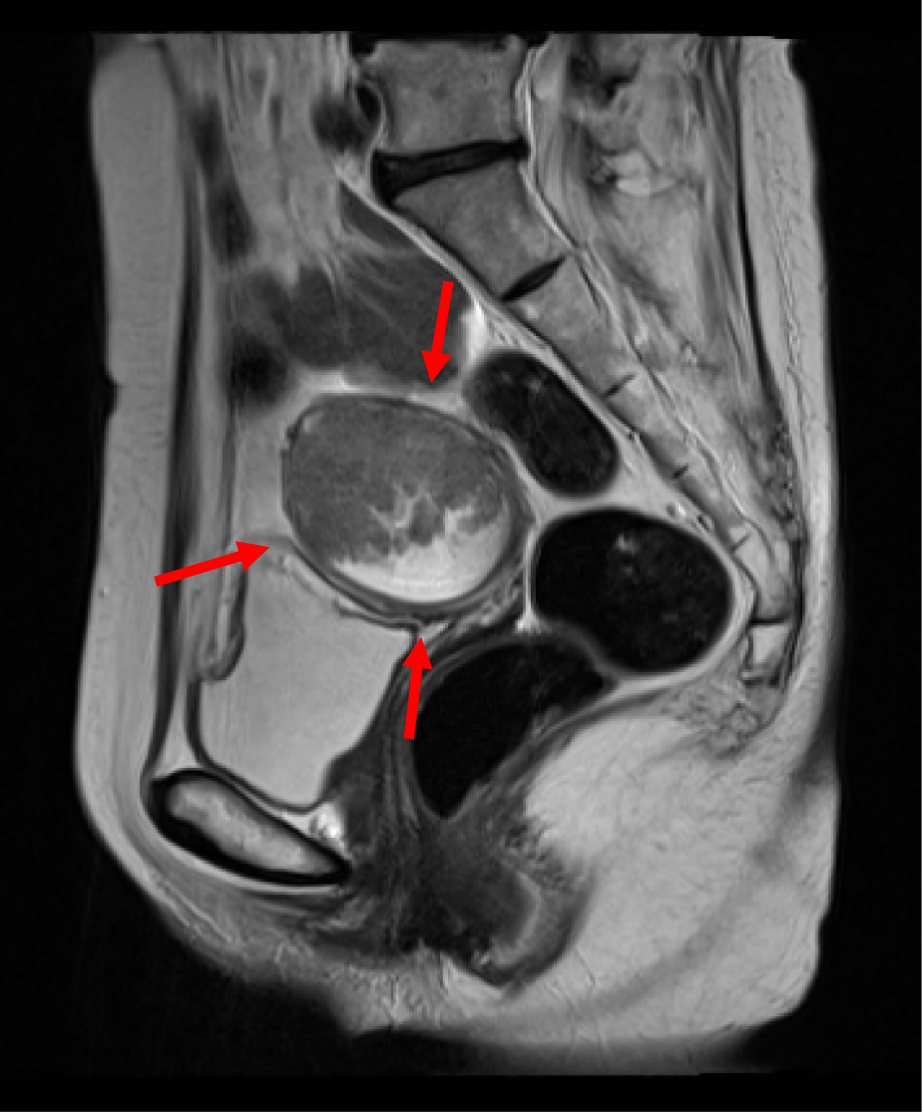
MRI shows invasion of the endometrial tumor into the outer half of the myometrium in the uterine corpus

**Fig. 2:**
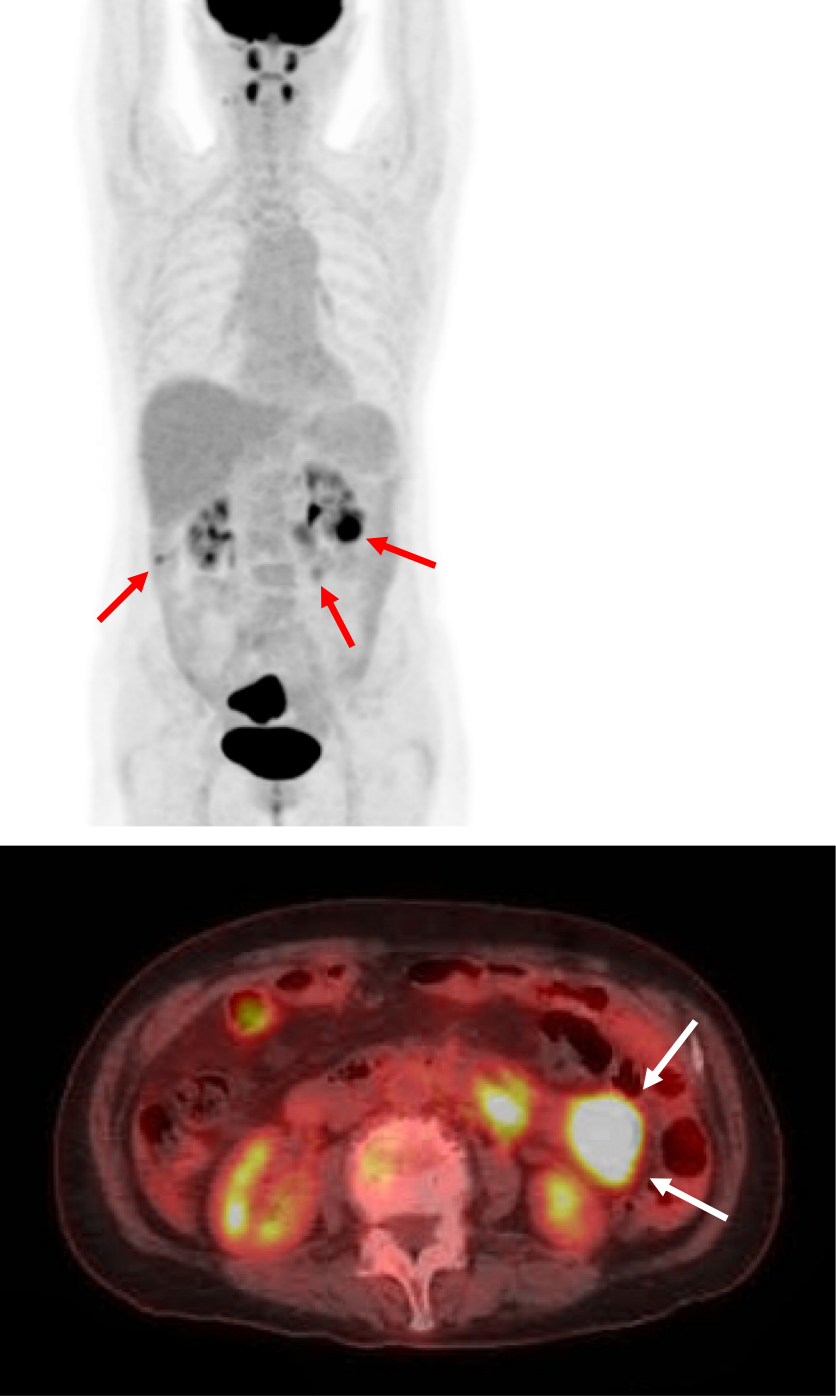
PET-CT shows intense focal FDG uptake in the endometrium and multiple massive lymphadenopathies involving the mesentery and para-aortic region

Pelvic ultrasound showed a mass, 5.6 cm × 3.8 cm in size, in the uterine corpus, without an adnexal mass. Endometrial biopsy revealed a grade 1 endometrioid adenocarcinoma. MRI showed invasion of the tumor into the outer half of the myometrium (Fig. 1). Abdominal CT showed para-aortic and atypical mesentery lymphadenopathy, which was suspected to be either a metastasis of the endometrial cancer or a malignant lymphoma. PET-CT showed intense focal FDG uptake in the endometrium and multiple massive lymphadenopathies involving the mesentery and para-aortic region (Fig. 2). Serum tumor markers were as follows: CA125 371.7 U/ml (≦ 35 U/ml) and CA19-9 98.5 U/ml (≦ 37 U/ml).

An abdominal hysterectomy with bilateral salpingo-oophorectomy, pelvic and para-aortic lymphadenectomy, partial omentectomy, and a mesentery lymph node biopsy for endometrial cancer were performed. Frozen section of the uterus was performed. It showed a G1 endometrioid carcinoma with an invasion of under one-half of the myometrium.

**Fig. 3:**
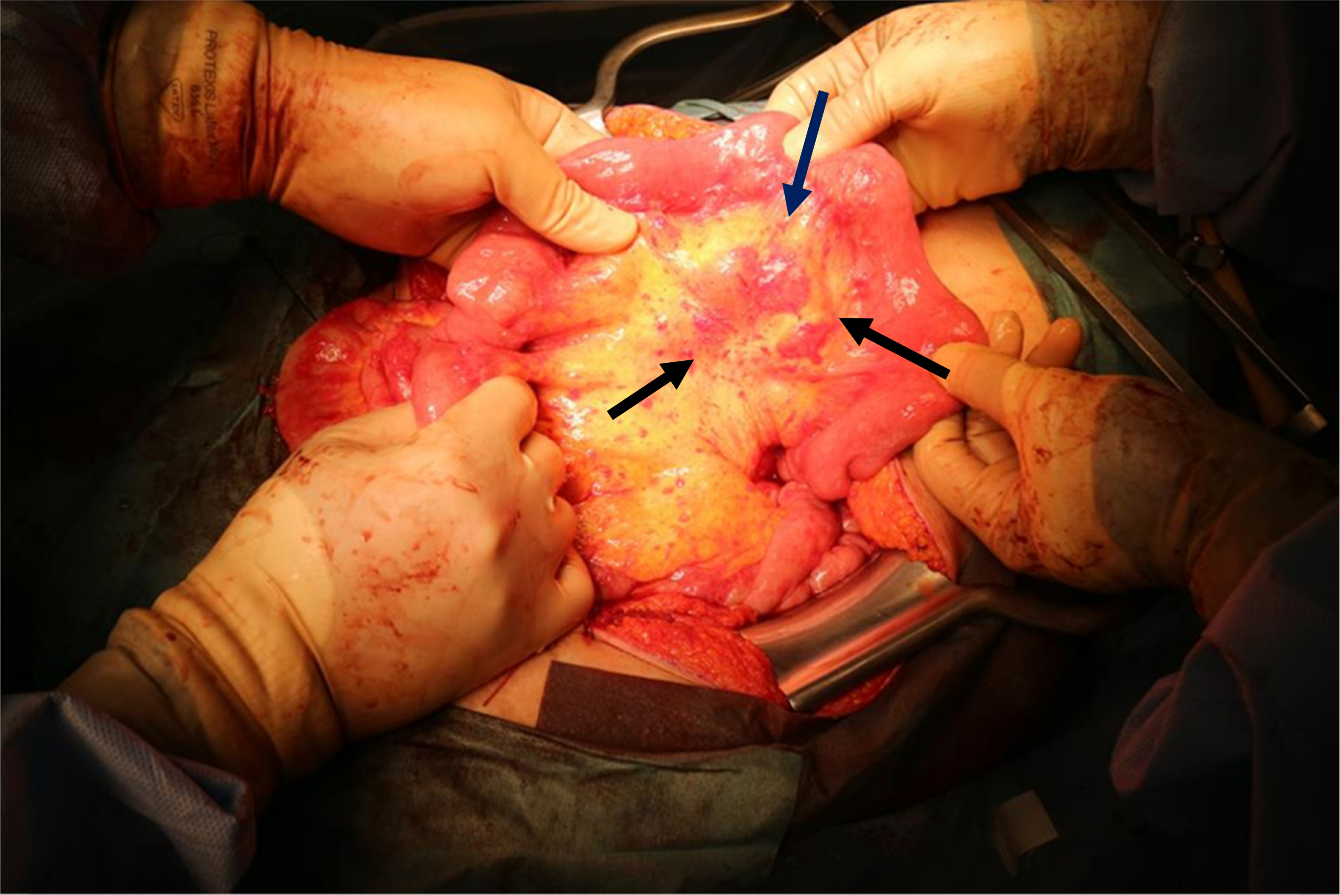
Patient undergoing endometrial surgery, with atypical mesenteric lymphadenopathy

**Fig. 4:**
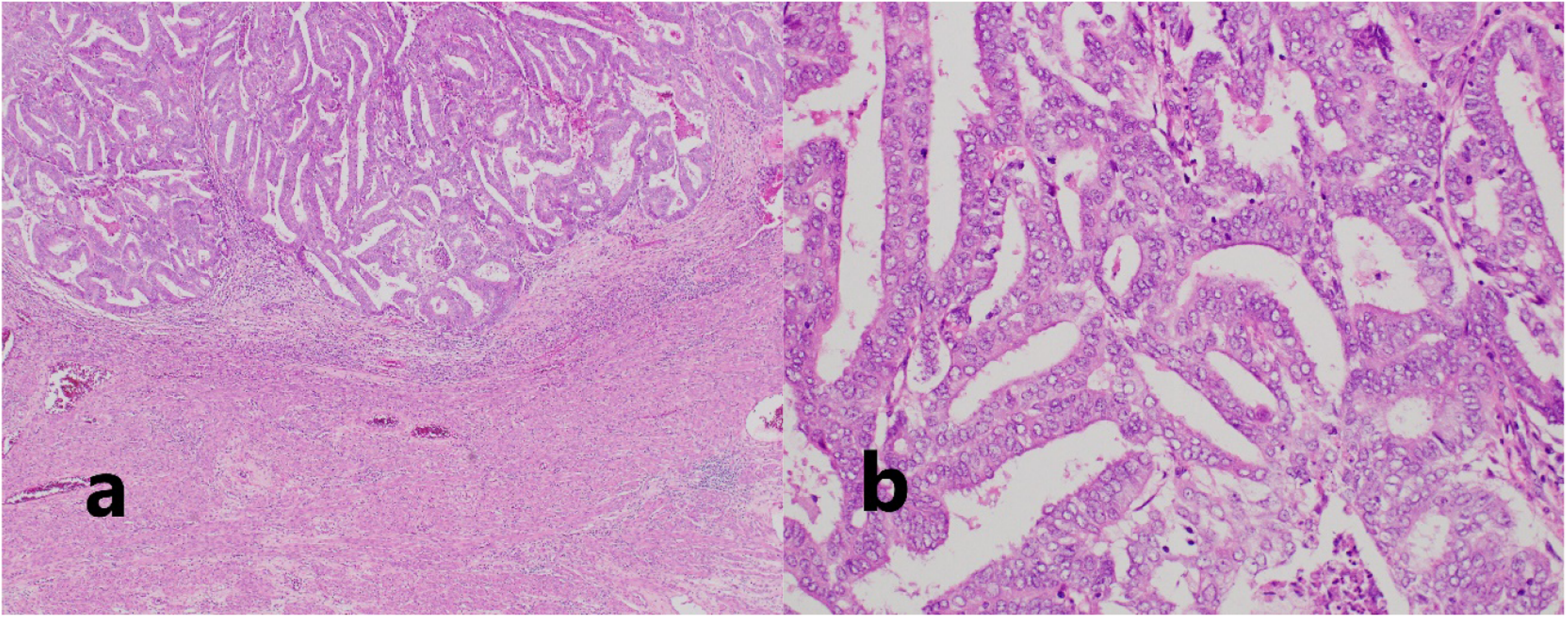
FIGO grade1 endometrioid endometrial carcinoma (H-E, **a** × 4, **b** × 20)

**Fig. 5:**
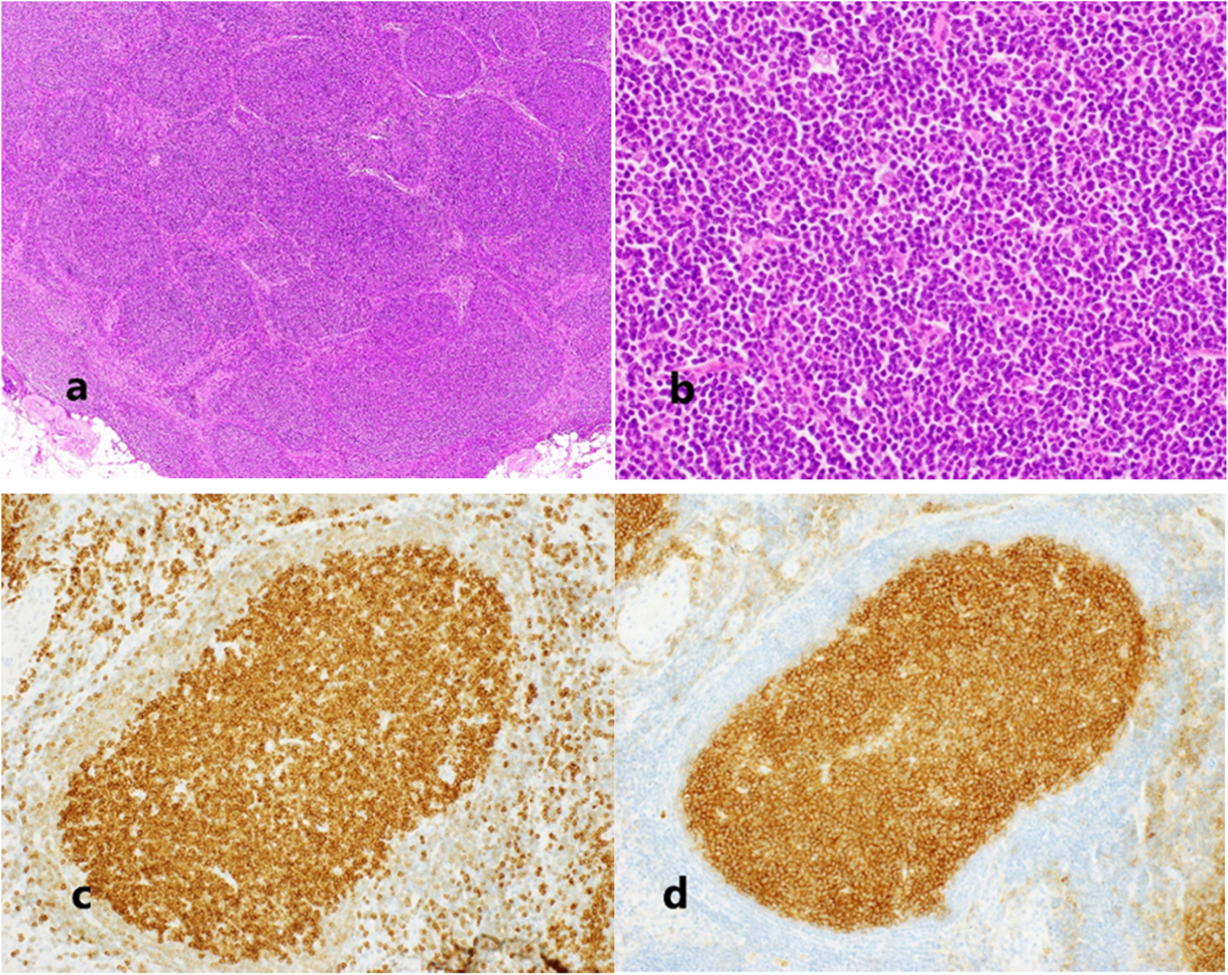
Mesentery lymph node showed distended follicles (H-E, **a** × 4, **b** × 40). The germinal center was positive for both Bcl-2 (**c**) and CD10 (**d**)

We performed a pelvic and para-aortic lymphadenectomy, as we could not rule out the involvement of a metastasis of the endometrial cancer. Because we strongly suspected a synchronous malignancy to be responsible for the mesentery lymphadenopathy, we performed a selective biopsy of the mesenteric lymph nodes (Fig. 3). The mesentery and para-aortic lymph nodes that were sent for frozen section analysis showed no metastasis of the endometrial cancer. As the preoperative imaging had indicated that the multiple mesenteric lymphadenopathy was unusual, we had previously discussed with our hematologists whether they could perform intra-operative flow cytometric analysis, which they usually performed in a post-operative clinical scenario. This procedure, including tissue dissection, antibody reaction, and flow cytometry, was expected to take approximately 60 min, so it was reasonable to attempt to perform it during surgery. Thus, in collaboration with our department of hematology, we conducted an intraoperative emergent four-color flow cytometry of the mesenteric lymphadenopathy tissue. Intraoperative flow cytometry analysis of the mesenteric lymph nodes indicated an abnormal κ/λ ratio of 0.17, resembling a B cell lymphoma. We diagnosed the patient as having an atypical mesentery adenopathy. Because, intraoperatively, we could find this mesenteric lymphadenopathy was derived from lymphoma, we avoided doing an unnecessary intestinal resection. The final pathological diagnosis was of an endometrioid carcinoma (G1, pT1aN0M0, FIGO2014: IA, ly0, v0), with a class IV peritoneal cytology from a synchronous follicular lymphoma (G1) (Figs. 4 and 5).

**Fig. 6:**
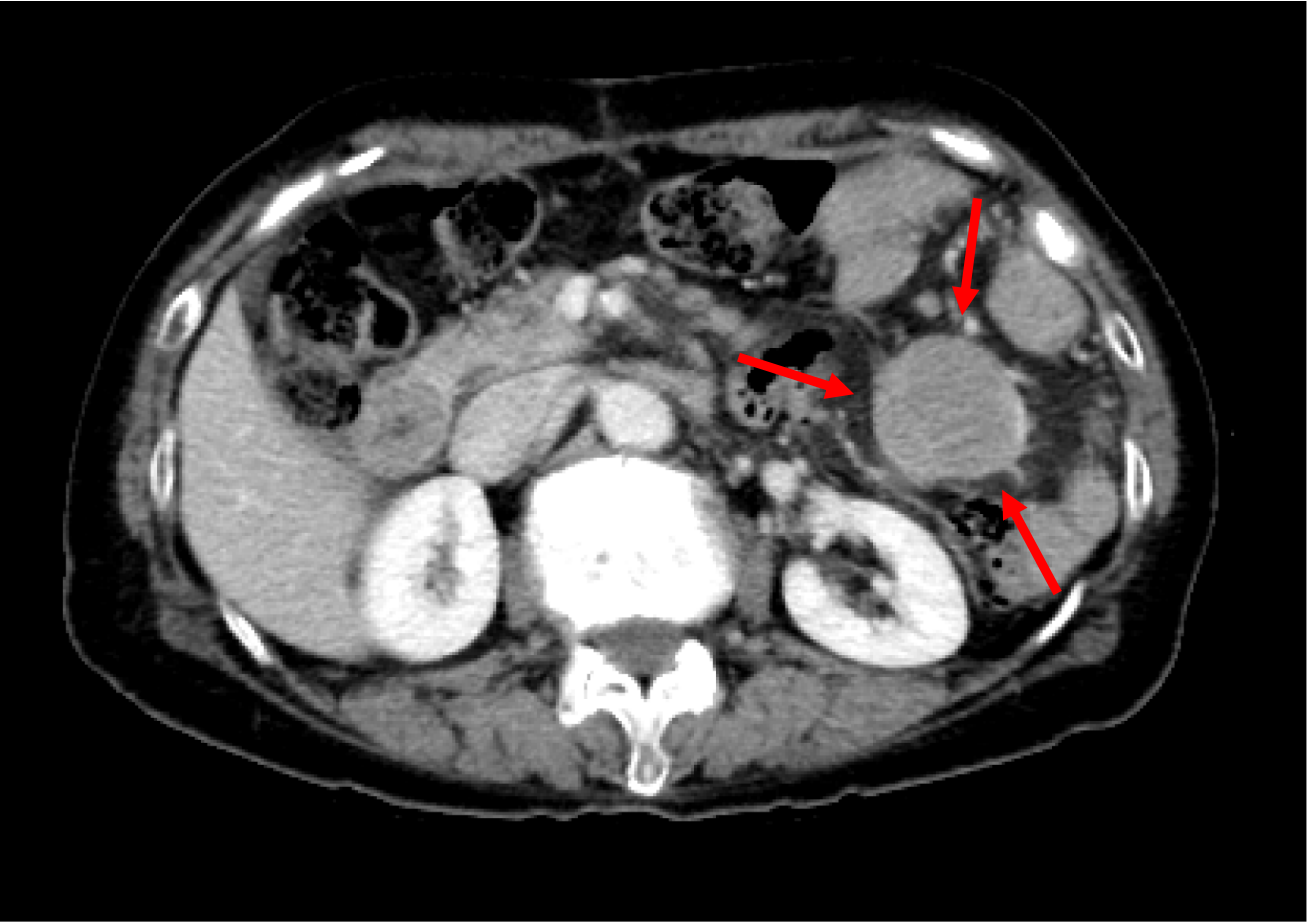
A CT scan shows progression of the residual lymphadenopathy three months after the operation

The mesentery lymph node showed diffuse back to back follicles. The follicles were composed of predominantly small- to medium-sized lymphoid cells with no tingible body macrophages. Immunohistochemical studies of the lymphoma were positive for CD20, Bcl-2, and CD10 and negative for CD3 and cyclinD1. The cytogenetic study of the mesentery lymph node showed a t(14;18)(q32;q21) chromosome translocation. The beta-2 microglobulin level at diagnosis was 2.0 mg/dl (0.8–1.8 mg/dl). A bone marrow biopsy specimen showed no evidence of overt involvement from the follicular lymphoma. Because of the lymphoma’s initial indolent clinical features, the patient was followed conservatively, without additional therapy. Three months after the operation, a CT scan showed a progression of the residual lymphadenopathy (Fig. 6), so rituximab monotherapy, with eight weekly infusions, was administrated. As of 18 months after surgery, the patient has shown no other evidence of recurrent disease.

## Discussion and conclusion

Multiple primary malignancies can and do occur in the same patient. Colon, ovarian, and breast cancers have been reported previously to be the malignancies most commonly associated as coexisting with endometrial cancer [11, 12], perhaps because they share certain common genetic predispositions.

Synchronous carcinoma and non-Hodgkin’s lymphoma have also been reported. The most common primary carcinomas associated with a synchronous non-Hodgkin’s lymphoma were in the colon, followed by the prostate, lung, breast, and stomach [13]. The sites for the lymphoma were varied. However, to our knowledge, there have been reported only five cases of the coexistence of a non-Hodgkin’s lymphoma of the retroperitoneal lymph nodes with endometrial cancer (Table 1) [5–9]. In all other cases, the presence of synchronous malignant lymphoma is an unexpected finding during surgery for endometrial cancer, and its diagnosis has been made only incidentally, after staging surgery. Since a synchronous malignant lymphoma with an endometrial cancer is so rare, it may be difficult to be aware of its possibility and thus to plan for an optimal treatment.

**Table 1:** Non-Hodgkin's lymphoma and endometrial cancer

		Endometrial cancer			Non-Hodgkin’s lymphoma				Follow-up since initial presentation	Refs.
Case no.	Age (years)	Histology	Stage	Treatment	Site	Histology	Lineage	Treatment		
1	72	Endometrioid	IIIA	ARH/BSO/PLND/PALND, RT	Pelvic/paraaortic lymph nodes	FL	B cell	None	No evidence of disease at 9 m	[4]
2	56	Endometrioid	IIIA	TAH/BSO/PLND	Uterus, ovary, and pelvic lymph nodes	FL, grade1	B cell	CT, RT	Died of NHL at 6 years	[6]
3	64	Endometrioid	IB	Cytoreductive surgery, CT, RT	Pelvic/paraaortic lymph nodes	FL	B cell	None	No evidence of disease at 9 months	[7]
4	58	Endometrioid	IIIC	TAH/BSO/PLND/PALND, CT, RT	Pelvic/paraaortic lymph nodes	CLL/SLL	B cell	None	NR	[8]
5	53	Endometrioid	NR	TAH/BSO/PLND/PALND	Pelvic/paraaortic lymph nodes	FL	B cell	NR	NR	[9]
6	67	Endometrioid	IA	TAH/BSO/PLND/PALND	Paraaortic/mesenteric lymph nodes	FL, grade1	B cell	CT	No evidence of disease at 18 m	Ours

*TAH* total abdominal hysterectomy, *BSO* bilateral salpingo-oophorectomy, *PLND* pelvic lymph node dissection, *PALND* para-aortic lymph node dissection, *ARH* abdominal radical hysterectomy, *NHL* non-Hodgkin’s lymphoma, *FL* follicular lymphoma, *CLL/SLL* chronic lymphocytic leukemia/small lymphocytic lymphoma, *CT* chemotherapy, *RT* radiation therapy, *NR* not reported

An accurate preoperative and intraoperative differentiation between a metastatic carcinoma and a lymphoma is often difficult. A confirming diagnosis of non-Hodgkin’s lymphoma is made by a biopsy combined with some combination of pathological examination, immunohistochemistry, flow cytometry, chromosome analysis, and gene analysis. In our case, distinguishing the lymphoma from the metastatic carcinoma during surgery was important in order to avoid an unnecessary intestinal resection.

As we strongly suspected, from the preoperative CT and intraoperative findings, that the atypical mesentery lymphadenopathy was a lymphoma, we had already planned to conduct frozen section and intraoperative flow cytometry analysis. However, a differential diagnosis by frozen section between a poorly differentiated carcinoma and a lymphoma can be difficult.

The alternative intraoperative assessment for lymphoma is flow cytometry. Since its introduction over three decades ago, flow cytometry has become a basic and rapid technique for the diagnosis and classification of hematological malignancies through evaluation of lymphoid B cell and T cell antigens and clonality assessment of the light chain. Flow cytometry sensitivity ranges from 75 to 99% and specificity from 87 to 100% [14–16]. There has been only one other report of the use of intraoperative flow cytometry, and it was during a neurosurgical procedure. Accurate intraoperative discrimination between primary central nervous system lymphoma and glioblastoma by frozen section is also sometimes difficult. Instead, DNA aneuploidy and S-phase status were evaluated by intraoperative flow cytometry. The accuracy of intraoperative flow cytometry was nearly equivalent to that of pathologic diagnosis using the permanent tumor specimen [17].

During surgery, intraoperative flow cytometry is useful for rapid differentiation between two tumors. In our case, the B cell lymphoma in the lymph nodes was diagnosed intraoperatively by our flow cytometry analysis, which showed an abnormal κ/λ ratio in the lymph nodes. Flow cytometry is thus highly useful for the intraoperative differentiation between metastatic carcinoma and lymphoma, leading to better decision-making within a relatively short time.

We diagnosed the coexistence of the lymphoma with the endometrial cancer intraoperatively using a multidisciplinary intraoperative assessment, which resulted in us avoiding an unnecessary intestinal resection. In a patient with a gynecological cancer, non-Hodgkin’s lymphoma may involve a multitude of adjacent sites. Although a rare event in endometrial cancer surgery, it is necessary to be alert to the possibility of a synchronous lymphoma in cases of unusual site adenopathy.

## Data Availability

All the data are available in the medical record.

## References

[1] Song L, Li Q, Yang K (2018). Three primary synchronous malignancies of the uterus, cervix, and fallopian tube: a case report. Medicine (Baltimore).

[2] Moertel CG, Dockerty MB, Baggenstoss AH (1961). Multiple primary malignant neoplasms. Cancer.

[3] Axelrod JH, Fruchter R, Boyce JG (1984). Multiple primaries among gynecologic malignancies. Gynecol Oncol.

[4] Carson HJ (1996). Unexpected synchronous non-Hodgkin’s lymphoma encountered during the treatment of a previously-diagnosed carcinoma: report of three cases. Leuk Lymphoma.

[5] Kostopoulos IS, Barbanis SB, Kaloutsi VD (2000). Synchronous occurrence of multiple malignant neoplasms in the uterus (adenocarcinoma of the endometrium, large B-cell lymphoma of the cervix). Pathol Res Pract.

[6] Vang R, Silva EG, Medeiros LJ, Deavers M (2000). Endometrial carcinoma and non-Hodgkin's lymphoma involving the female genital tract: a report of three cases. Int J Gynecol Pathol.

[7] Lee HB, Park JC, Lee YS (2011). Unexpected synchronous follicular lymphoma of paraaortic and pelvic lymph nodes in a patient with endometrial carcinoma: a case report. Eur J Gynaecol Oncol.

[8] Dłuski D, Lewkowicz D, Leszczynska-Gorzelak B (2018). An unusual coexistence of chronic lymphocytic leukemia/small lymphocytic lymphoma with endometrioid-type endometrial cancer in a 58-year-old woman: a case study with literature review. Case Rep Oncol.

[9] Yoon S-N (2018). Follicular lymphoma mimicking metastatic nodes on the F-18 FDG PET/CT and MRI for staging of endometrial cancer. Nucl Med Mol Imaging.

[10] Alduaij A, Hansen K, Zhang C (2010). Primary follicular lymphoma of the fallopian tube found incidentally in a patient treated for endometrial carcinoma: a case report. Diagn Pathol.

[11] Annegers JF, Malkasian GD (1981). Patterns of other neoplasia in patients with endometrial carcinoma. Cancer.

[12] Tangjitgamol S, Khunnarong J, Srijaipracharoen S (2015). Synchronous and metachronous malignancy in endometrial cancer patients treated in a tertiary care center of Thailand. J Gynecol Oncol.

[13] Mariani A, Cha SS, Bergstralh EJ (2010). Referral and ascertainment bias in patients with synchronous and metachronous endometrial malignancy. Eur J Gynaecol Oncol.

[14] Peluso AL, Leni A, Mignogna C (2016). Lymph node fine-needle cytology: beyond flow cytometry. Acta Cytologica.

[15] Cozzolino I, Rocco M, Villani G (2016). Lymph node fine-needle cytology of non-Hodgkin lymphoma: diagnosis and classification by flow cytometry. Acta Cytologica.

[16] Craig FE, Foon KA (2008). Flow cytometric immunophenotyping for hematologic neoplasms. Blood.

[17] Koriyama S, Nitta M, Shioyama T (2018). Intraoperative flow cytometry enables the differentiation of primary central nervous system lymphoma from glioblastoma. World Neurosurg.

